# Examining the Prevalence of Anterior Cruciate Ligament Injuries on Artificial Turf Surfaces Compared to Natural Grass Surfaces in Athletes: A Scoping Review

**DOI:** 10.7759/cureus.63770

**Published:** 2024-07-03

**Authors:** Jared N Kushner, Tomas Swickley, Rishiraj Bandi, Jayson Lian, Michelle K Knecht, Lea Sacca

**Affiliations:** 1 Department of Population Health and Social Medicine, Florida Atlantic University Charles E. Schmidt College of Medicine, Boca Raton, USA; 2 Department of Orthopaedic Surgery, Montefiore Medical Center, Wakefield Campus, Bronx, USA

**Keywords:** soccer, football, natural turf, artificial turf, anterior cruciate ligament (acl)

## Abstract

The anterior cruciate ligament (ACL) is commonly injured in sports such as American football and soccer. It is currently unknown if ACL injuries are more prevalent on natural grass or artificial turf fields. The purpose of this scoping review is to analyze research studies evaluating the effect of the playing surface on the prevalence of ACL injuries. We hypothesize that athletes face a greater risk of suffering ACL injuries while playing on artificial turf compared to natural grass. Our team conducted a comprehensive literature review by screening three databases (PubMed, Embase, and Cochrane) that comprised a wide range of peer-reviewed articles on ACL injuries suffered on natural grass and artificial turf surfaces. Inclusion criteria consisted of epidemiological and cohort studies published after 1990 that were written in English and focused on athletes ranging in skill level from youth to professional. Exclusion criteria consisted of biomechanical studies, review articles, and papers that focused on injuries of structures other than the ACL. Bias was assessed with the MINORS criteria. Results were presented by injury rates, calculated ratios, and confidence intervals. The final analysis included nine papers published in peer-reviewed journals. Three of nine papers found that ACL injuries are more likely to occur on artificial turf than on natural grass. Three papers found that there is no difference in the prevalence of ACL injuries between surfaces and one paper stated that ACL injuries are more likely on natural grass than artificial turf. Two papers did not report confidence intervals for ratios comparing injury rates between playing surfaces. There is no consensus in the current literature regarding the prevalence of ACL injuries on artificial turf versus natural grass surfaces. The primary limitation of this study was that the papers used a variety of methods to compare rates of ACL injuries on artificial and natural surfaces, making comparisons between the nine papers difficult.

## Introduction and background

The anterior cruciate ligament (ACL) is crucial for knee stability during both exercise and daily movement [1]. The ACL’s function as secondary resistance to internal rotation of the tibia prevents translation of the tibia upon full extension of the knee, while also lowering the torque strain of the knee upon flexion [2]. Such safeguards are necessary not only for walking, but also for running, pivoting, stomping, and twisting of the knee, making the ACL prone to damage from overuse and accidents in sports [3]. A major concern for athletes is the impact playing surfaces have on the increasing prevalence of ACL injuries [4].

Artificial surfaces began appearing in American professional sports in the 1960s [5]. These surfaces’ benefits, including durability, lack of required maintenance, and consistency across different types of weather, have led many parks, schools, and professional sports teams to favor synthetic surfaces over natural grass [6]. As of 2020, over 13,000 synthetic turf fields were in use across the United States [7]. Since artificial fields have grown in popularity, concerns over the safety of athletes on these surfaces have risen, especially when compared to natural grass [4].

Numerous biomechanical experiments have studied the relationship between playing surfaces and ACL injuries. Dowling et al. found that surfaces with a greater coefficient of friction predispose athletes to make a sidestep-cutting motion with less knee flexion and more knee valgus [8]. Additionally, a cadaveric study by Drakos et al. tested the differences in ACL strain between various combinations of shoes and surfaces [9]. Their results indicated that cleats on natural grass produced less strain on the ACL than cleats or turf shoes on artificial turf when placed under an axial load or on internal rotation [9]. Furthermore, a team at the University of Virginia performed a study on the mechanical interactions of playing surfaces and the force exerted by professional athletes [10]. The study demonstrated that grass surfaces allow for tearing of the grass and that there is greater horizontal force production on artificial surfaces [10]. Despite being of interest to the public, each individual study demonstrated no definitive answer as to whether artificial turf posed a significantly higher risk of injury [10].

Furthermore, a search of the present clinical literature demonstrates conflicting information regarding the risk of athletes suffering ACL injuries on artificial surfaces as compared to natural grass. For instance, a study by Howard et al. demonstrated a statistically significant increase in ACL injuries on grass [11], while Dragoo et al. showed a greater rate of injury on artificial surfaces [12].

Currently, there are no recent reviews of the literature that focus on the association between ACL injuries and playing surfaces. The purpose of this scoping review is to fill the existing gap in the literature by identifying relevant studies on this topic and providing an assessment of the epidemiological information available on the relationship between field types and rates of ACL injuries. We hypothesize that the present literature will predominantly demonstrate a greater risk of ACL injuries on artificial surfaces than on natural surfaces.

## Review

Methods

The present study utilized the 2020 PRISMA-ScR checklist throughout our review process (Appendix Tables 1, 2). Arksey and O’Malley’s (2005) York Methodology guided the review. This framework is composed of five steps: (1) identifying research questions; (2) searching for relevant studies; (3) selecting studies relevant to the research questions; (4) charting the data; and (5) collating, summarizing, and reporting the results. The utilization of both the PRISMA-ScR checklist and the Arksey and O’Malley framework allows for a systematic approach when sharing the findings relevant to the selected research questions.

Step 1: Identify Research Questions

The primary research question for our study was: Are ACL injuries more prevalent on natural grass or artificial turf playing surfaces?

Step 2: Search for Relevant Studies

The literature search was performed by three independent reviewers (JK, TS, RB) in January and February 2023, with the aid of the university librarian (MK). The keywords (MeSH terms for PubMed, Emtree terms for Embase, and Keywords for Cochrane) included in the search were “grass”, “natural grass”, “poaceae”, “natural field”, “artificial turf”, “synthetic turf”, “playing surface”, “artificial playing surface”, “anterior cruciate ligament injury”, “ACL injury”, “football”, “soccer”, “rugby”, and “lacrosse”. Literature searches were performed through PubMed, Embase, and Cochrane. The Rayyan platform was used to compile the papers our team identified from the first screening and allowed each reviewer to blindly vote to include or discard each paper.

Inclusion and exclusion criteria were set prior to the commencement of the literature search. Inclusion criteria included epidemiologic and cohort studies that were published from 1990 and onward, were written in English, and had athletes with skill levels ranging from youth to professional levels of their respective sports. Exclusion criteria included biomechanical studies that did not evaluate a sample group for relevant injury statistics, review articles with no observational statistics done on their own, and papers that measured statistics on injuries that did not specifically damage the ACL.

Step 3: Select Studies Relevant to the Research Question

**Figure 1:**
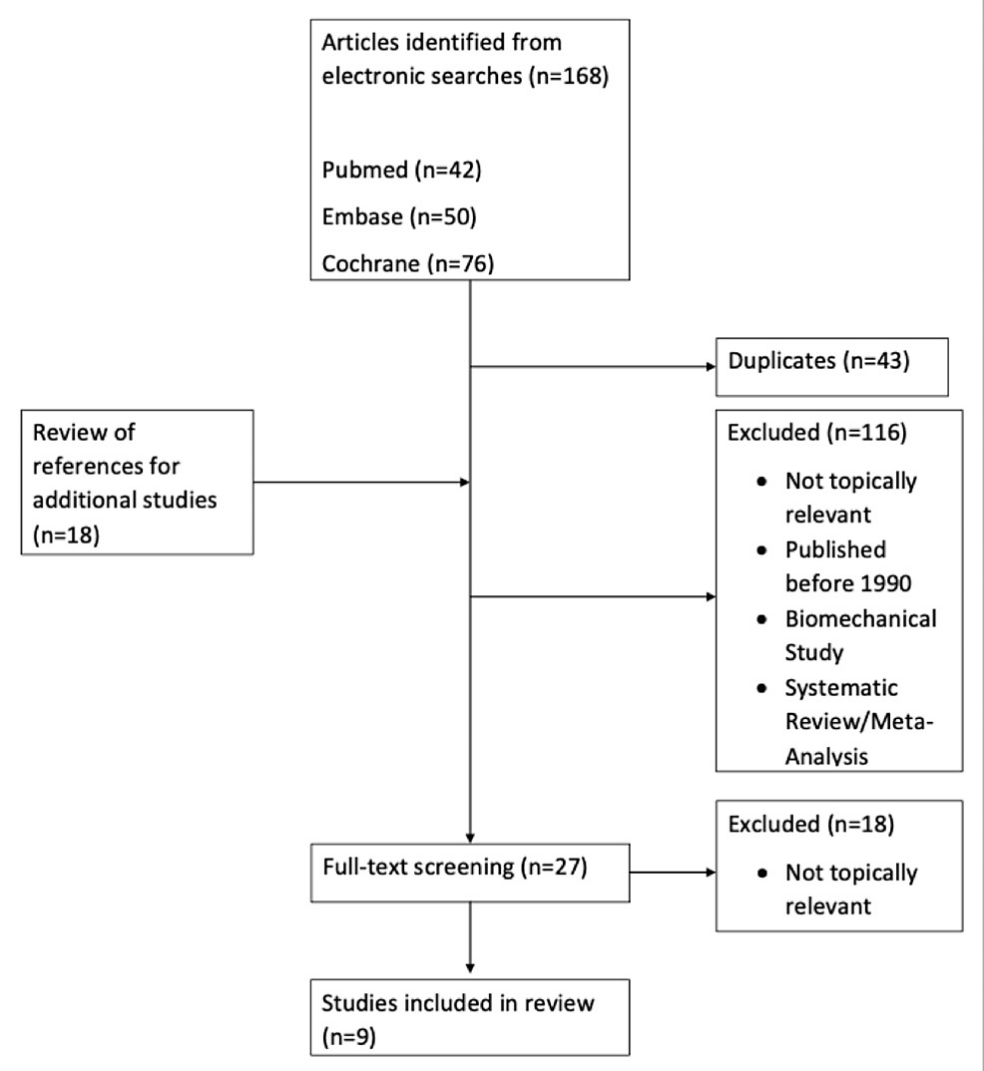
PRISMA Flow Chart of the Study Selection Process

As displayed in Figure 1, the search performed by our team produced 168 papers to be evaluated. Forty-three duplicate papers were removed. The reference section of each of the remaining 125 papers was also searched to identify additional studies to include, yielding 18 more papers. The review process then began with the three reviewers voting on whether to include each paper. Papers were evaluated based on the inclusion and exclusion criteria as well as their relevance to the research question. Papers that received two or three votes proceeded to the next round of review. The first round of review was to evaluate the topic of the papers based on their titles which eliminated 98 papers, yielding 44 to be evaluated based on their abstracts. Sixteen papers were eliminated in the abstract review stage, leaving 27 to be read in their entirety. Nine papers were chosen in this final round to be included in our analysis. All nine of the final papers were evaluated for bias by the three reviewers utilizing the MINORS criteria.

Step 4: Chart the Data

The data was charted by tabulating study characteristics and results. In Table 1, level of play, injury of interest, years of investigation, and method of analysis were noted. Table 2 includes the injury rates on each surface, the rate ratio reported by each paper, and confidence intervals.

**Table 1:** Study Characteristics ACL: Anterior Cruciate Ligament; NFL: National Football League; NCAA: National Collegiate Athletic Association.

Primary Author	Study Design	Sample Size	Target Population	Publication Year	Injury of Interest	Level of Play	Years Under Investigation	Method of Analysis
Dodson et al. [13]	Descriptive Epidemiological Study	219 ACL Injuries	Professional Football Athletes	2016	ACL Injuries	NFL	2010-2013	Rate of Injury
Dragoo et al. [14]	Descriptive Epidemiological Study	318 ACL Injuries	Collegiate Football Athletes	2012	ACL Injuries	NCAA	2004-2009	Rate Ratios and Injury Rates
Hagglund and Waldén [15]	Cohort Study	96 Acute Knee Injuries	Youth Soccer Players	2016	ACL Injuries	Youth Girls Soccer	2009	Rate Ratios
Hershman et al. [16]	Descriptive Epidemiological Study	1528 Knee Sprains	Professional Football Athletes	2012	Lower Extremity Injuries	NFL	2000-2009	Incidence Density Ratio
Howard et al. [17]	Cohort Study	3449 ACL Injuries	Collegiate Soccer Athletes	2020	ACL Injuries	NCAA	2004-2014	Incidence Rate Ratio
Loughran et al. [18]	Descriptive Epidemiological Study	2460 Knee Injuries	Collegiate Football Athletes	2019	ACL Injuries	NCAA	2004-2014	Rate Ratios
Ngatuvai et al. [19]	Descriptive Epidemiological Study	1039 ACL Injuries	High School Football and Soccer Athletes	2022	ACL Injuries	High School	2007-2019	Injury Proportion Ratio
Powell and Schootman [20]	Descriptive Epidemiological Study	10,326 Injuries	Professional Football Athletes	1992	Knee Injuries	NFL	1980-1989	Incidence Density Ratio
Scranton et al. [21]	Descriptive Epidemiological Study	78 Non-Contact ACL Injuries	Professional Football Athletes	1997	ACL Injuries	NFL	1989-1993	Incidence Density Ratio

**Table 2:** Study Results *Statistically significant greater rate of injuries on artificial turf. **Statistically significant greater rate of injuries on natural grass. ACL: Anterior Cruciate Ligament; NFL: National Football League; NCAA: National Collegiate Athletic Association; AEs: Athletic Exposures; CI: Confidence Interval.

Primary Author/Type of Sport	Sources of Data	Measure of ACL Injury Frequency (Incidence/Prevalence)	Rate of ACL Injuries		Ratio
			Artificial Playing Surface	Grass Playing Surface	
Football					
Dodson (2016) [13]	USA Today NFL Games Database	Incidence	0.053 Per Team-Game	0.050 Per Team-Game	Not Available
Dragoo (2012) [14]	NCAA Injury Surveillance System	Incidence	1.73 Per 10,000 AEs	1.24 Per 10,000 AEs	Rate Ratio: 1.39, 95% CI (1.11-1.75)*
Hershman (2012) [16]	NFL Injury Surveillance System	Incidence	0.069 Per Team-Game	0.041 Per Team Game	Incidence Density Ratio: 1.67, 95% CI (1.30-2.15)*
Loughran (2019) [18]	NCAA Injury Surveillance System	Incidence	Competitions: 10.51 Per 10,000 AEs; Practice: 0.99 Per 10,000 AEs	Competition: 8.92 Per 10,000 AEs; Practice: 0.92 Per 10,000 AEs	Rate Ratio: Competition: 1.18, 95% CI (0.92-1.52); Practice: 1.08, 95% CI (0.84-1.39)
Ngatuvai (2022) [19]	High School Reporting Information Online Surveillance System	Prevalence	74,620 Per 100,000 AEs	122,654 Per 100,000 AEs	Injury Proportion Ratio: 1.23, 95% CI (1.03-1.47)*
Powell (1992) [20]	NFL Athletic Trainers	Incidence	0.02 Per Team-Game	0.02 Per Team-Game	Incidence Density Ratio: 1.10, 95% CI (0.73-1.54)
Scranton (1997) [21]	NFL Injury Surveillance System	Incidence	0.1085 Per 1,000 AEs	0.0569 Per 1,000 AEs	Not Available
Soccer					
Hagglund (2016) [15]	Randomized Control Trial Cohort	Incidence	0.08 Per 1,000 hours	0.08 Per 1,000 hours	Rate Ratio: 1.0, 95% CI (0.23-4.29)
Howard (2020) [17]	NCAA Injury Surveillance System (ISS) Database	Incidence	0.92 Per 10,000 AEs	1.16 Per 10,000 AEs	Incidence Rate Ratio: 1.26, 95% CI (1.14-1.38)**
Ngatuvai (2022) [19]	High School Reporting Information Online Surveillance System	Prevalence	71,877 Per 100,000 AEs	104,028 Per 100,000 AEs	Injury Proportion Ratio: Males: 1.65, 95% CI (0.99-2.75); Females: 1.53, 95% CI (1.08-2.16)*

Step 5: Collate, Summarize, and Report the Results

Although the reviews utilized various ratios to analyze injury rates between artificial turf and natural turf, the majority of studies provided p-values or confidence intervals, which were used to determine the statistical significance of the differences in injury rates between surfaces.

Results

The initial study extraction yielded 168 papers from PubMed (n=42), Embase (n=50), and Cochrane (n=76). Duplicate papers (n=43) were excluded. A review of the reference sections of the papers yielded additional (n=18) studies. Following the sequential review of titles, abstracts, and complete manuscripts, nine papers were retained for analysis, all of which were published between 1990 and 2022. Three of the papers were published within the last five years. Study designs included cohort studies (n=2) and descriptive epidemiological studies (n=7). The sample sizes of injuries addressed in the studies ranged from 78 injuries to 3448 injuries. Methods of analysis include incidence density ratio (n=3), rate ratios (n=3), injury rate (n=1), injury proportion ratio (n=1), and incidence rate ratio (n=1).

Priority Populations

The reviewed papers focused on various groups of athletes. The populations can be grouped by the sport the athletes play and by the level of athletic participation. Sports included American football (n=7) and soccer (n=3), including one paper that analyzed injuries in both sports. Levels of participation included professional (n=4), collegiate (n=3), high school (n=1), and youth (n=1). All four papers discussing ACL injuries in professional athletes focused on American football players. Two of the papers discussing injuries in collegiate athletes focused on American football and one focused on soccer. The paper that discusses high school athletes included data on American football and soccer players. The paper focused on youth athletes solely discussed soccer players.

Databases Accessed

Seven of the nine papers we reviewed collected their data from publicly accessible databases, including the NFL Injury Surveillance System (n=2), USA Today NFL Games Database (n=1), NCAA Injury Surveillance System (n=3), and the National High School Sports-Related Injury Surveillance Study (n=1). The remaining paper that focused on NFL athletes collected data via team athletic trainers. The paper that focused on youth soccer collected their own data from local athletes.

Injury Frequency

The compiled list of articles used for this review is shown in Table 1. Each paper presented injury frequency in terms of injury per athlete exposure, per team game, or per hour of play. The authors of the included articles then used these epidemiological findings to calculate measures of association including incidence density ratios, rate ratios, injury rates, injury proportion ratios, and incidence rate ratios to determine if one playing surface poses a greater risk of ACL injury than the other. Although the reviews had varying modes of analysis to compare the injury rates of the ACL between artificial turf and natural turf, most of the studies provided insight into the significance of their calculated ratio by providing 95% confidence intervals.

The results of the papers we reviewed are presented in Table 2. Three of the papers demonstrated an outright difference in injury rates between playing surfaces, with injuries on artificial turf being more common than injuries on natural grass [14,16,19]. Ngatuvai et al. displayed an increased risk on artificial turf for female soccer players but not for their male counterparts [19]. Three of the papers demonstrated no statistically significant difference between injury rates on the two surfaces [15,18,20]. Additionally, Dodson et al. did not provide a specific p-value or confidence interval for their data but did state that there was no significant difference between the injury rates [13]. Only one paper, by Howard et al., demonstrated a statistically significant difference between injury rates on artificial turf and natural grass, where ACL injuries were more common on natural grass [17].

For American football specifically, three of the seven studies presented a statistically significant incidence of ACL injuries occurring on artificial turf compared to natural turf [14,16,19]. Three of the remaining four found no significant difference between the surfaces [13,18,20]. The one remaining study that focused on American football, by Scranton et al., did not provide a p-value or confidence interval and did not state whether or not the data was statistically significant [21]. However, the incidence of ACL injuries on artificial turf was greater than the incidence of injuries on grass [21]. Of the papers that investigated soccer, only Ngatuvai et al. displayed an increased risk of ACL injuries on turf compared to grass, although as previously stated this finding was only significant for female athletes [19]. For the male soccer players in Ngatuvai et al. and all of the athletes in Hagglund et al., there was no statistically significant difference in ACL injuries between groups [15,19]. Howard et al. presented data showing a statistically significant increased risk of ACL injuries on natural grass than artificial turf for soccer players [17].

When stratifying by level of play, only one of the four studies that investigated professional sports showed a significantly higher rate of ACL injury on artificial turf [16]. One of the three studies focused on NCAA sports shared similar results [14]. One of the studies that investigated collegiate athletes presented a significantly greater rate of ACL injuries on natural grass [17]. The study by Ngatuvai et al. that focused on high school athletes showed a significantly greater risk of ACL injuries on turf than grass for football players and for women’s soccer players [19]. Hagglund et al. focused on youth athletes and did not find a statistically significant difference between surfaces [15].

Of the two papers that were published before 2010, only one provided a p-value of their data, which showed that there was not a statistically significant difference between injury rates on grass and turf [20]. Of the papers published after 2010, three report significantly more ACL injuries on turf than grass [14,16,19]. The validity of each of the studies was confirmed using the MINORS assessment, which proved successful in proving the efficacy and findings of each of the studies. Based on the included articles, scores from the MINORS criteria ranged from 10 to 14.

Discussion

This scoping review sought to compare the rates of ACL injuries on natural grass fields and artificial turf fields. This topic is of great concern to athletes and has caused much controversy in recent years, particularly in the National Football League. The current literature lacks reviews that assess ACL injuries across multiple sports and multiple levels of play and evaluates papers published within the last five years. The present paper aims to fill this gap and enhance our knowledge of how playing surface impacts ACL injuries, with the goal of improving player safety.

Three studies reported statistically significant differences in injury rates across playing surfaces, with more injuries occurring on turf than grass. Additionally, three studies reported no difference in injury rates between surfaces and one study reported a greater rate of ACL injuries on natural grass than turf. Therefore, there is no consensus in the present literature as to whether or not there is a greater risk of ACL tears on artificial surfaces. The results of this review allow us to definitively conclude that there is not a greater risk of ACL injuries on natural grass than on artificial turf.

Three of the seven papers that discuss American football demonstrate a greater risk of ACL injuries on artificial than natural surfaces. Meanwhile, only one of the three papers that discuss soccer has similar findings. The study by Ngatuvai et al. showed a significantly greater risk of ACL injuries on turf than on grass for soccer players; however, this risk was only present in female soccer players [19]. One of the studies focusing on soccer players showed no significant difference between injury rates on the two surfaces and another determined that injuries are more common on natural grass than artificial turf. After stratifying the data by sport we can conclude that it is more likely that football players, as compared to soccer players, have greater risk of ACL injuries on artificial turf than natural grass. The data on soccer players is incredibly inconsistent, preventing us from drawing a definitive conclusion. However, the data on football players also showed mixed results as two of the studies provided confidence intervals demonstrating no significant difference in injury rates and another did not provide their statistics but did state that there was no difference between the groups. From these results, we can state that football players may be at a greater risk of ACL injury when playing on turf fields but the evidence is not resounding enough for this finding to be conclusive. Alternatively, we can conclude that football players are not more likely to suffer ACL injuries on natural grass than on turf.

Only one of four papers that discuss professional sports demonstrated an increased risk of ACL injuries on artificial turf than natural grass, whereas one of the three papers on NCAA sports reported a greater risk on turf than grass. The one paper on high school sports demonstrated inconsistent results that vary by sport. The paper on youth athletes showed no difference in surfaces. Stratifying the data by level of play did not yield any conclusions about risks varying by skill level. However, it is plausible to assume that higher levels of play allow athletes access to playing surfaces of greater quality. Future research may consider collecting more extensive data and comparing the risk of ACL injury between various levels of play to draw conclusions about the impact of the quality of the playing surfaces.

Neither of the two studies published before 2010 demonstrated an increased risk of ACL injuries on either playing surface, whereas three of the papers published after 2010 showed a greater risk of injuries on turf than grass and one showed a greater risk on grass than turf. While the limited data prior to 2010 makes it difficult to form any conclusions, it is important to consider how the evolution of artificial turf technology could alter injury rates. For example, first-generation turf had issues with seams that caused irregularities in the field and were concerning for athlete safety [4]. Current turf technology has allowed for improvements to its implementation such as infill that allows for increased planarity across the playing surface [4]. In theory, a surface that is more even is safer for athletes playing sports upon them. However, a review of the present literature may indicate that the risk of ACL injuries on turf is greater on newer surfaces.

From youth leagues to professional organizations, a significant amount of athletes practice and compete on artificial fields. According to the Synthetic Turf Council, approximately half of the teams in the National Football League play on synthetic turf [22]. Many collegiate programs, especially in northern regions with harsher winters, are also converting to artificial turf fields for their aforementioned benefits. With this widespread utilization of turf across multiple sports and levels of play, it is worth considering the implications these fields may have on injury prevalence.

Through this review of the current literature, it is still unclear as to whether artificial turf or natural grass has a higher potential for injury risk in competitive sports. Further studies are necessary to establish injury trends on these surfaces and account for a wider array of sports and types of playing surfaces in reference to the prevalence of ACL injuries.

This comprehensive review of the present literature discusses the most up-to-date data on ACL injuries and their relationship to playing surfaces. Analysis of the most present data is crucial for this topic, considering that artificial turf technology is rapidly evolving and the risk of injury may change with newer generations of artificial playing surfaces. Furthermore, this review covered papers that focus on multiple sports and multiple levels of play, allowing for analysis that is generalizable to a wide range of athletes. The main limitation of this paper was that there were no measures to standardize the findings of the selected articles. Therefore, we could only comment on the results of each study based on its own method of analysis, allowing us to only compare the final qualitative results of the study, and not their quantitative measures. Additionally, the majority of the studies we analyzed retrieved their data from publically accessible databases. We believe that more cohort studies would strengthen the available data.

## Conclusions

The goal of our scoping review was to review the current literature discussing the differences in risk of ACL injuries between natural turf and artificial turf. The primary conclusion from our review is that there is no universal consensus on whether there is a greater risk of ACL injuries on natural grass or artificial turf. This study may stimulate future research to further elucidate the risk factors for ACL injury, including playing surface.

## Appendices

**Table 3:** PRISMA 2020 Main Checklist Source: Page MJ, McKenzie JE, Bossuyt PM, Boutron I, et al. The PRISMA 2020 statement: an updated guideline for reporting systematic reviews. MetaArXiv. 2020, September 14. DOI: 10.31222/osf.io/v7gm2. For more information, visit www.prisma-statement.org

Topic	No.	Item	Location where item is reported
TITLE			
Title	1	Identify the report as a systematic review.	Page 2
ABSTRACT			
Abstract	2	See the PRISMA 2020 for Abstracts checklist	
INTRODUCTION			
Rationale	3	Describe the rationale for the review in the context of existing knowledge.	Page 2
Objectives	4	Provide an explicit statement of the objective(s) or question(s) the review addresses.	Page 2
METHODS			
Eligibility criteria	5	Specify the inclusion and exclusion criteria for the review and how studies were grouped for the syntheses.	Page 2
Information sources	6	Specify all databases, registers, websites, organisations, reference lists and other sources searched or consulted to identify studies. Specify the date when each source was last searched or consulted.	Page 2
Search strategy	7	Present the full search strategies for all databases, registers and websites, including any filters and limits used.	Page 2
Selection process	8	Specify the methods used to decide whether a study met the inclusion criteria of the review, including how many reviewers screened each record and each report retrieved, whether they worked independently, and if applicable, details of automation tools used in the process.	Page 2
Data collection process	9	Specify the methods used to collect data from reports, including how many reviewers collected data from each report, whether they worked independently, any processes for obtaining or confirming data from study investigators, and if applicable, details of automation tools used in the process.	Page 2
Data items	10a	List and define all outcomes for which data were sought. Specify whether all results that were compatible with each outcome domain in each study were sought (e.g. for all measures, time points, analyses), and if not, the methods used to decide which results to collect.	Page 2
	10b	List and define all other variables for which data were sought (e.g. participant and intervention characteristics, funding sources). Describe any assumptions made about any missing or unclear information.	Page 2
Study risk of bias assessment	11	Specify the methods used to assess risk of bias in the included studies, including details of the tool(s) used, how many reviewers assessed each study and whether they worked independently, and if applicable, details of automation tools used in the process.	Page 2
Effect measures	12	Specify for each outcome the effect measure(s) (e.g. risk ratio, mean difference) used in the synthesis or presentation of results.	Page 3
Synthesis methods	13a	Describe the processes used to decide which studies were eligible for each synthesis (e.g. tabulating the study intervention characteristics and comparing against the planned groups for each synthesis (item 5)).	Page 2
	13b	Describe any methods required to prepare the data for presentation or synthesis, such as handling of missing summary statistics, or data conversions.	N/A
	13c	Describe any methods used to tabulate or visually display results of individual studies and syntheses.	Page 3
	13d	Describe any methods used to synthesize results and provide a rationale for the choice(s). If meta-analysis was performed, describe the model(s), method(s) to identify the presence and extent of statistical heterogeneity, and software package(s) used.	N/A
	13e	Describe any methods used to explore possible causes of heterogeneity among study results (e.g. subgroup analysis, meta-regression).	N/A
	13f	Describe any sensitivity analyses conducted to assess robustness of the synthesized results.	N/A
Reporting bias assessment	14	Describe any methods used to assess risk of bias due to missing results in a synthesis (arising from reporting biases).	Page 2
Certainty assessment	15	Describe any methods used to assess certainty (or confidence) in the body of evidence for an outcome.	Page 4
RESULTS			
Study selection	16a	Describe the results of the search and selection process, from the number of records identified in the search to the number of studies included in the review, ideally using a flow diagram.	Page 3
	16b	Cite studies that might appear to meet the inclusion criteria, but which were excluded, and explain why they were excluded.	N/A
Study characteristics	17	Cite each included study and present its characteristics.	Page 5
Risk of bias in studies	18	Present assessments of risk of bias for each included study.	Page 7
Results of individual studies	19	For all outcomes, present, for each study: (a) summary statistics for each group (where appropriate) and (b) an effect estimate and its precision (e.g. confidence/credible interval), ideally using structured tables or plots.	Page 6
Results of syntheses	20a	For each synthesis, briefly summarise the characteristics and risk of bias among contributing studies.	Page 3-4
	20b	Present results of all statistical syntheses conducted. If meta-analysis was done, present for each the summary estimate and its precision (e.g. confidence/credible interval) and measures of statistical heterogeneity. If comparing groups, describe the direction of the effect.	N/A
	20c	Present results of all investigations of possible causes of heterogeneity among study results.	N/A
	20d	Present results of all sensitivity analyses conducted to assess the robustness of the synthesized results.	N/A
Reporting biases	21	Present assessments of risk of bias due to missing results (arising from reporting biases) for each synthesis assessed.	N/A
Certainty of evidence	22	Present assessments of certainty (or confidence) in the body of evidence for each outcome assessed.	Page 6
DISCUSSION			
Discussion	23a	Provide a general interpretation of the results in the context of other evidence.	Page 7-8
	23b	Discuss any limitations of the evidence included in the review.	Page 8
	23c	Discuss any limitations of the review processes used.	Page 8
	23d	Discuss implications of the results for practice, policy, and future research.	Page 8
OTHER INFORMATION			
Registration and protocol	24a	Provide registration information for the review, including register name and registration number, or state that the review was not registered.	N/A
	24b	Indicate where the review protocol can be accessed, or state that a protocol was not prepared.	N/A
	24c	Describe and explain any amendments to information provided at registration or in the protocol.	N/A
Support	25	Describe sources of financial or non-financial support for the review, and the role of the funders or sponsors in the review.	Page 8
Competing interests	26	Declare any competing interests of review authors.	Page 8
Availability of data, code and other materials	27	Report which of the following are publicly available and where they can be found: template data collection forms; data extracted from included studies; data used for all analyses; analytic code; any other materials used in the review.	Page 6

**Table 4:** PRISMA Abstract Checklist Source: Page MJ, McKenzie JE, Bossuyt PM, et al. The PRISMA 2020 statement: an updated guideline for reporting systematic reviews. MetaArXiv. 2020, September 14. DOI: 10.31222/osf.io/v7gm2. For more information, visit www.prisma-statement.org

Topic	No.	Item	Reported?
TITLE			
Title	1	Identify the report as a systematic review.	Yes
BACKGROUND			
Objectives	2	Provide an explicit statement of the main objective(s) or question(s) the review addresses.	Yes
METHODS			
Eligibility criteria	3	Specify the inclusion and exclusion criteria for the review.	Yes
Information sources	4	Specify the information sources (e.g. databases, registers) used to identify studies and the date when each was last searched.	Yes
Risk of bias	5	Specify the methods used to assess risk of bias in the included studies.	Yes
Synthesis of results	6	Specify the methods used to present and synthesize results.	Yes
RESULTS			
Included studies	7	Give the total number of included studies and participants and summarise relevant characteristics of studies.	Yes
Synthesis of results	8	Present results for main outcomes, preferably indicating the number of included studies and participants for each. If meta-analysis was done, report the summary estimate and confidence/credible interval. If comparing groups, indicate the direction of the effect (i.e. which group is favoured).	Yes
DISCUSSION			
Limitations of evidence	9	Provide a brief summary of the limitations of the evidence included in the review (e.g. study risk of bias, inconsistency and imprecision).	Yes
Interpretation	10	Provide a general interpretation of the results and important implications.	Yes
OTHER			
Funding	11	Specify the primary source of funding for the review.	No
Registration	12	Provide the register name and registration number.	No
